# Coronary Artery Disease in End-Stage Renal Disease Patients on Dialysis With Hepatic Dysfunction: Prevalence and Predictors

**DOI:** 10.7759/cureus.105271

**Published:** 2026-03-15

**Authors:** Sughra Chandio, Muhammad Ramzan Danish, Alizay Mukhtar, Muhammad Sohail Akhtar Ansari, Syed Muhammad Hassan Bukhari, Zarmeen Mubarak, Muhammad Areeb Khawaja

**Affiliations:** 1 Family Medicine, Shah Medical Centre, Karachi, PAK; 2 General Surgery, Pakistan Navy Hospital PNS Shifa, Karachi, PAK; 3 Internal Medicine, Imtiaz medical Centre, Lahore, PAK; 4 Cardiology, Dr. Faisal Masood Teaching Hospital, Sargodha, PAK; 5 Internal Medicine, Sunderland Royal Hospital, London, GBR; 6 Medicine, King's College Hospital, London, GBR; 7 Health Department, Project Management Unit, Maryam Nawaz Health Clinics, Lahore, PAK; 8 General and Emergency Medicine, Social Security Basic Health Unit, Punjab Employees Social Security Institution (PESSI), Muzaffargarh, PAK

**Keywords:** cardiac risk factors and prevention, coronary artery disease, end stage renal disease (esrd), hepatic dysfunction, predictors

## Abstract

Background: Patients with end-stage renal disease (ESRD) undergoing maintenance dialysis are at markedly increased risk of coronary artery disease (CAD). The presence of hepatic dysfunction is frequently observed in this population and may be associated with additional metabolic and inflammatory disturbances that influence cardiovascular outcomes.

Objective: This study assessed the prevalence of CAD and identified its significant predictors among ESRD patients with concurrent hepatic impairment.

Methods: A retrospective observational study was conducted at Mayo Hospital, Lahore, from August 2024 to August 2025, reviewing medical records of 369 ESRD patients undergoing maintenance hemodialysis with documented hepatic dysfunction. Demographic characteristics, comorbidities, dialysis-related parameters, laboratory findings, and hepatic profiles were extracted. Hepatic dysfunction severity was classified using the Child-Pugh system. CAD was diagnosed based on ≥50% coronary artery stenosis on angiography, regional wall motion abnormalities on echocardiography suggestive of ischemia, or positive noninvasive ischemia testing.

Results: The mean age of the participants was 57.8 ± 11.6 years, and 58.5% were male. The overall prevalence of CAD was 38.7% (n = 143). Among CAD-positive patients, 59 (41.3%) had angiographic stenosis ≥50%, 48 (33.6%) demonstrated regional wall motion abnormalities on echocardiography, and 36 (25.2%) had positive noninvasive ischemia tests. Patients with CAD were significantly older (61.2 ± 10.7 vs. 55.9 ± 11.8 years, p < 0.001) and had a higher prevalence of diabetes mellitus and dyslipidemia. Dialysis duration was longer in CAD-positive patients (4.9 ± 2.4 vs. 3.8 ± 1.9 years, p < 0.001). Multivariable regression analysis identified older age (AOR 1.04, 95% CI 1.02-1.07), diabetes mellitus (AOR 1.89, 95% CI 1.20-2.98), prolonged dialysis duration (AOR 1.28, 95% CI 1.12-1.46), elevated CRP (AOR 1.75, 95% CI 1.08-2.84), and moderate-to-severe hepatic dysfunction (AOR 2.14, 95% CI 1.31-3.48) as independent predictors of CAD, while albumin demonstrated a protective association (AOR 0.72, 95% CI 0.54-0.96).

Conclusion: CAD is highly prevalent among ESRD patients undergoing maintenance dialysis with concurrent hepatic dysfunction. Age, diabetes mellitus, dialysis duration, systemic inflammation, and severity of hepatic impairment significantly contribute to CAD risk in this population.

## Introduction

Coronary artery disease (CAD) remains one of the leading challenges in cardiovascular medicine, particularly in patients with end-stage renal disease (ESRD) on dialysis. Patients in this group are extremely heterogeneous, and the observed cardiovascular mortality is greater than that of any other population [1]. Although diabetes, hypertension, and dyslipidemia are traditionally considered to be the main contributors to cardiovascular risk, ESRD is particularly complex. Inflammation, endothelial dysfunction, oxidative stress, vascular calcification, and uremia-related metabolic dysregulation accelerate atherosclerosis in ways that conventional models often overlook. The most complex and tragic of these models is ESRD [2]. Along with its life-saving properties, dialysis exacerbates hemodynamic instability, myocardial strain, and rapid electrolyte changes, increasing the risk of ischemia. The dysfunction becomes more complicated when there is concomitant hepatic dysfunction [3]. Liver disease increases cardiovascular risk by disrupting lipid metabolism, promoting inflammation [4], altering vasoactive regulation, and creating an unstable balance between procoagulant and anticoagulant pathways, thereby amplifying systemic cardiovascular stress [5].

Due to this dual burden of renal and hepatic dysfunction, it is extremely hard to predict with significance the rapid escalation of CAD risk. It is becoming increasingly evident that traditional models predicting CAD are even more inaccurate in patients with ESRD and hepatic dysfunction. Predictive models such as the Framingham risk score and others fall short in populations with advanced vascular calcification, chronic inflammatory syndromes, anemia of chronic disease, and hemodynamic instability, in addition to severe metabolic instability [6]. Many patients on dialysis fall into the group of patients suffering from atypical or silent ischemia due to autonomic neuropathy, shifts in blood volume, and impaired nociceptive pain response. This contributes to a prolonged time to diagnosis and aggravates the situation clinically. When hepatic dysfunction is also present, this adds even more complexity to the situation, as symptoms of fatigue, shortness of breath, or volume overload might be mistaken for decompensated cirrhosis rather than resulting from cardiac dysfunction [7].

From a pathophysiological standpoint, ESRD and liver dysfunction are both directed along the same harmful pathways: oxidative stress, chronic inflammation, endothelial damage, imbalance of nitric oxide, and alterations of calcium-phosphate metabolism. These factors interact in such a way as to generate a synergistic effect rather than contribute to risk in an additive manner [8]. Patients suffering from chronic liver disease show evidence of greater than the expected rate of subclinical atherosclerosis and impaired coronary flow reserve, along with microvascular dysfunction [9]. The global prevalence of cardiovascular disease (CVD) remains a foremost problem in public health, drastically increasing incidences of morbidity and mortality, and in patients who also present with chronic kidney disease (CKD), the problem is even more severe [10]. For patients with CKD who receive hemodialysis (HD) treatment, the probability of suffering adverse cardiovascular events is even more pronounced, owing to the impact of traditional and non-traditional risk factors [11]. There is a recognized relationship between CKD and CVD, and the progression of CKD is known to increase the risk of CVD. Adverse cardiovascular events are, unfortunately, an even more severe problem in the dialysis population, being a primary cause of their morbidity and mortality, which is disproportionate to their nondialysis counterparts [12].

This study assessed the prevalence of CAD and identified its significant predictors among ESRD patients with concurrent hepatic impairment.

## Materials and methods

This retrospective observational study was conducted at Mayo Hospital, Lahore, from August 2024 to August 2025. A total of 369 ESRD patients undergoing maintenance hemodialysis were included. A non-probability consecutive sampling approach was used, in which all patient records meeting the eligibility criteria during the specified timeframe were included.

Inclusion criteria

Patients aged 18 years or older with a confirmed diagnosis of ESRD undergoing maintenance hemodialysis for at least three months were eligible. Only those with documented hepatic dysfunction based on elevated liver enzymes, bilirubin abnormalities, hypoalbuminemia, prolonged INR, or radiological evidence of chronic liver disease were included. Hepatic dysfunction was defined and graded using the Child-Pugh classification system, which incorporates serum bilirubin, serum albumin, prothrombin time/INR, ascites, and hepatic encephalopathy. Patients were categorized as: Class A (mild hepatic dysfunction); Class B (moderate hepatic dysfunction); Class C (severe hepatic dysfunction).

Exclusion criteria

Patients were excluded if they had acute kidney injury instead of ESRD, acute hepatic failure, sepsis-related hepatic dysfunction, congenital heart disease, non-atherosclerotic cardiomyopathies, or incomplete medical records lacking essential clinical or laboratory data.

Data collection

Data were extracted using a structured proforma from electronic and paper-based hospital records. Collected variables included demographic characteristics, duration of ESRD, dialysis duration and frequency, type of vascular access, and comorbidities such as diabetes mellitus and hypertension. Laboratory parameters included renal profile, liver function tests, inflammatory markers (C-reactive protein), lipid profile, and calcium-phosphate product. To ensure temporal consistency, liver function parameters and cardiac assessments were considered only if performed within the same six-month clinical window. Cardiac evaluation was based on available electrocardiography, echocardiography, stress testing, and coronary angiography. Coronary artery disease (CAD) was defined as ≥50% stenosis in a major coronary artery on angiography, regional wall motion abnormalities suggestive of ischemia on echocardiography, or documented myocardial ischemia on noninvasive stress testing.

Statistical analysis

Data were analyzed using IBM SPSS Statistics for Windows, Version 26 (Released 2019; IBM Corp., Armonk, New York). Continuous variables were expressed as mean ± standard deviation, while categorical variables were presented as frequencies and percentages. Bivariate analysis was performed using independent t-tests for continuous variables and chi-square tests for categorical variables. Variables with clinical relevance and those with p < 0.10 in bivariate analysis were entered into a multivariable logistic regression model to identify independent predictors of CAD. Adjusted odds ratios (AOR) with 95% confidence intervals were calculated. Multicollinearity was assessed using variance inflation factors (VIF), and variables with VIF >5 were evaluated for model stability. Model discrimination was assessed using the area under the receiver operating characteristic curve, and calibration was evaluated using the Hosmer-Lemeshow goodness-of-fit test. A p-value <0.05 was considered statistically significant.

## Results

Data were collected from 369 patients. The mean age of the cohort was 57.8 ± 11.6 years, with males comprising 58.5% of the population. Hypertension was the most prevalent comorbidity, affecting 82.1% of the patients, followed by diabetes mellitus at 63.4% and dyslipidemia at 38.2%. The mean duration of hemodialysis was 4.3 ± 2.1 years. Most patients were dialyzing through an arteriovenous fistula (71.0%), while 22.5% had tunneled catheters and 6.5% used AV grafts. Laboratory trends showed substantial hepatic impairment: 62.3% had elevated ALT or AST, 54.7% had hypoalbuminemia, and 28.4% demonstrated prolonged INR. Based on hepatic dysfunction severity, nearly half of the patients (47.7%) had mild impairment, 35.0% had moderate dysfunction, and 17.3% had severe hepatic involvement (Table 1).

**Table 1:** Baseline Characteristics of ESRD Patients With Hepatic Dysfunction (N = 369) SD: standard deviation, AV: arteriovenous, ALT: alanine aminotransferase, AST: aspartate aminotransferase, INR: international normalised ratio, ESRD: end-stage renal disease.

Variables	Values
Age (years), mean ± SD	57.8 ± 11.6
Sex, n (%)	
Male	216 (58.5%)
Female	153 (41.5%)
Comorbidities, n (%)	
Hypertension	303 (82.1%)
Diabetes mellitus	234 (63.4%)
Dyslipidemia	141 (38.2%)
Dialysis duration (years), mean ± SD	4.3 ± 2.1
Type of vascular access, n (%)	
AV fistula	262 (71.0%)
Tunneled catheter	83 (22.5%)
AV graft	24 (6.5%)
Liver function abnormalities, n (%)	
Elevated ALT/AST	230 (62.3%)
Hypoalbuminemia	202 (54.7%)
Prolonged INR	105 (28.4%)
Severity of hepatic dysfunction, n (%)	
Mild	176 (47.7%)
Moderate	129 (35.0%)
Severe	64 (17.3%)

CAD was identified in 143 patients, yielding an overall prevalence of 38.7%. Among those with CAD, 59 patients (41.3%) demonstrated ≥50% stenosis on coronary angiography, 48 patients (33.6%) showed regional wall motion abnormalities on echocardiography, and 36 patients (25.2%) had positive findings on noninvasive ischemia testing. CAD-positive patients were older, with a mean age of 61.2 ± 10.7 years compared to 55.9 ± 11.8 years in CAD-negative individuals. The burden of CAD was significantly higher in patients with diabetes mellitus, with 48.5% affected, and in hypertensive patients, where 41.8% showed evidence of CAD (Table 2).

**Table 2:** Prevalence and Patterns of Coronary Artery Disease (CAD) (N = 369) CAD diagnosis was based on coronary angiography, echocardiography demonstrating regional wall motion abnormalities consistent with ischemia, or positive non-invasive ischemia testing. Laboratory values used for CAD association analyses represent the mean of the two most recent pre-dialysis measurements recorded within three months prior to cardiac assessment. Blood samples for dialysis-related biochemical parameters were collected pre-dialysis during midweek sessions to minimize post-dialysis variability. Bivariate analyses were exploratory; only clinically relevant variables or those with p < 0.10 were considered for multivariable regression modeling. Multicollinearity among covariates was assessed using variance inflation factors before final model inclusion. CAD: coronary artery disease, SD: standard deviation.

CAD parameter	n (%)
Overall CAD prevalence, n (%)	143 (38.7%)
Evidence of CAD, n (%)	
Angiographic stenosis ≥50%	59 (41.3%)
Regional wall motion abnormalities	48 (33.6%)
Positive non-invasive ischemia testing	36 (25.2%)
Age comparison, mean ± SD	
CAD present: Mean age (years)	61.2 ± 10.7
CAD absent: Mean age (years)	55.9 ± 11.8
CAD in diabetics, n (%)	113/234 (48.5%)
CAD in hypertensives, n (%)	127/303 (41.8%)

Patients with CAD were significantly older (61.2 ± 10.7 vs. 55.9 ± 11.8 years, p < 0.001) and had a higher prevalence of diabetes mellitus (79.0% vs. 53.5%, p = 0.01). Dyslipidemia was also more common in the CAD group (47.6% vs. 32.3%, p = 0.03). Dialysis duration was notably longer in patients with CAD (4.9 ± 2.4 vs. 3.8 ± 1.9 years, p < 0.001), indicating that prolonged exposure to hemodialysis-related stress may contribute to CAD development. Serum albumin levels were significantly lower in the CAD group (2.8 ± 0.5 g/dL vs. 3.1 ± 0.6 g/dL, p = 0.002), while elevated CRP was more frequent among CAD-positive individuals (70.6% vs. 52.7%, p = 0.01). Severity of hepatic dysfunction was strongly associated with CAD, with 25.9% of CAD patients having severe hepatic involvement compared to 11.9% in the CAD-absent group (p = 0.004) (Table 3).

**Table 3:** Bivariate Analysis of Factors Associated With CAD (N = 369) Continuous variables were analyzed using an independent-samples t-test. Categorical variables were analyzed using the chi-square test of independence. A p-value < 0.05 was considered statistically significant.

Variable	CAD present (n = 143)	CAD absent (n = 226)	Effect size (95% CI)	p-value
Age (years)	61.2 ± 10.7	55.9 ± 11.8	Mean difference: 5.3 (2.9–7.7)	<0.001
Male sex, n (%)	87 (60.8)	129 (57.1)	OR: 1.16 (0.74–1.82)	0.51
Diabetes mellitus, n (%)	113 (79.0)	121 (53.5)	OR: 3.21 (1.95–5.29)	0.01
Dyslipidemia, n (%)	68 (47.6)	73 (32.3)	OR: 1.91 (1.22–2.98)	0.03
Dialysis duration (years)	4.9 ± 2.4	3.8 ± 1.9	Mean difference: 1.1 (0.6–1.6)	<0.001
Albumin (g/dL)	2.8 ± 0.5	3.1 ± 0.6	Mean difference: −0.3 (−0.4 to −0.1)	0.002
Elevated CRP (>10 mg/L), n (%)	101 (70.6)	119 (52.7)	OR: 2.15 (1.35–3.42)	0.01
Severe hepatic dysfunction, n (%)	37 (25.9)	27 (11.9)	OR: 2.57 (1.44–4.58)	0.004

Increasing age remained a significant predictor, with an adjusted odds ratio (AOR) of 1.04 per year (95% CI: 1.02-1.07, p < 0.001). Diabetes mellitus nearly doubled CAD risk (AOR: 1.89, p = 0.005), while prolonged dialysis duration significantly increased the likelihood of CAD (AOR: 1.28 per year, p < 0.001). Elevated CRP, reflecting systemic inflammation, was also independently associated with higher CAD risk (AOR: 1.75, p = 0.02). Moderate to severe hepatic dysfunction had a strong association with CAD (AOR: 2.14, p = 0.002), confirming the role of hepatic impairment in cardiovascular vulnerability (Table 4).

**Table 4:** Multivariable Logistic Regression for Independent Predictors of CAD AOR: adjusted odds ratio, CRP: C-reactive protein, CAD: coronary artery disease.

Variable	Adjusted odds ratio (AOR)	95% Confidence interval	p-value
Age (per year increase)	1.04	1.02–1.07	<0.001
Diabetes mellitus	1.89	1.20–2.98	0.005
Dialysis duration (per year)	1.28	1.12–1.46	<0.001
Elevated CRP	1.75	1.08–2.84	0.02
Moderate–severe hepatic dysfunction	2.14	1.31–3.48	0.002
Albumin level (protective)	0.72	0.54–0.96	0.03

CAD-positive patients had significantly lower hemoglobin levels (9.3 ± 1.1 vs. 9.8 ± 1.2 g/dL, p = 0.01) and higher urea levels (144.1 ± 38.5 vs. 136.4 ± 35.2 mg/dL, p = 0.04). Disturbances in mineral metabolism were prominent, with CAD patients exhibiting higher phosphate levels (6.3 ± 1.2 vs. 5.8 ± 1.1 mg/dL, p = 0.002) and elevated calcium-phosphate products (51.0 ± 10.6 vs. 47.4 ± 9.3, p = 0.006), both known contributors to vascular calcification. Inflammatory burden was greater in CAD patients, reflected by significantly elevated CRP levels (23.6 ± 8.4 vs. 18.9 ± 7.6 mg/L, p < 0.001). Nutritional status also differed, with lower albumin levels among CAD patients (2.8 ± 0.5 vs. 3.1 ± 0.6 g/dL, p = 0.002) (Table 5).

**Table 5:** Comparison of Laboratory and Dialysis-Related Parameters Between Patients With and Without CAD (N = 369) Continuous variables were analyzed using an independent-samples t-test. Categorical variables were analyzed using the chi-square test of independence. A p-value < 0.05 was considered statistically significant. Kt/V refers to dialysis adequacy, where K = dialyzer clearance of urea, t = dialysis time, and V = volume of distribution of urea in the patient. CAD: coronary artery disease, CRP: C-reactive protein.

Parameter	CAD present (n = 143)	CAD absent (n = 226)	Test statistic	p-value
Hemoglobin (g/dL)	9.3 ± 1.1	9.8 ± 1.2	t = –2.56	0.01
Serum creatinine (mg/dL)	8.9 ± 2.4	8.6 ± 2.2	t = 1.10	0.27
Urea (mg/dL)	144.1 ± 38.5	136.4 ± 35.2	t = 2.06	0.04
Calcium (mg/dL)	8.1 ± 0.7	8.3 ± 0.6	t = –2.15	0.03
Phosphate (mg/dL)	6.3 ± 1.2	5.8 ± 1.1	t = 3.20	0.002
Calcium-phosphate product	51.0 ± 10.6	47.4 ± 9.3	t = 2.76	0.006
Lipid profile abnormality, n (%)	96 (67.1%)	113 (50.0%)	χ² = 9.70	0.002
CRP (mg/L)	23.6 ± 8.4	18.9 ± 7.6	t = 5.24	<0.001
Albumin (g/dL)	2.8 ± 0.5	3.1 ± 0.6	t = –3.17	0.002
Dialysis frequency				
Twice weekly	89 (62.2%)	142 (62.8%)	χ² = 0.01	0.91
Thrice weekly	54 (37.8%)	84 (37.2%)		
Kt/V (dialysis adequacy)	1.09 ± 0.22	1.14 ± 0.25	t = –2.33	0.02
Interdialytic weight gain (kg)	3.1 ± 1.0	2.7 ± 0.9	t = 2.98	0.003

## Discussion

This retrospective study illustrated the considerable frequency of coronary artery disease in patients with end-stage renal disease on maintenance dialysis with concurrent hepatic impairment. CAD was noted in almost 40% of the sample and emphasizes the disproportionate cardiovascular risk associated with renal and hepatic dysfunction. This prevalence has also been noted in previous works that recorded CAD in dialysis patients between 30% and 50%, reinforcing that the conventional cardiovascular risk paradigm fails to elucidate the true extent of cardiovascular disease burden in ESRD patients.

Older age was identified as the most significant independent predictor of CAD, which coincides with literature demonstrating accelerated vascular senescence within the dialysis population [13]. Uremia-associated endothelial dysfunction, chronic inflammatory state, and vascular calcification tend to occur and progress more severely in the ESRD population, making even middle-aged individuals from that cohort incongruently older cardiovascularly. This study also illustrated the strong association of diabetes mellitus with CAD, further confirming the added burden of glucose dysregulation in the uremic state. Diabetic ESRD patients develop more extensive and calcified coronary lesions, greater microvascular dysfunction, higher rates of silent ischemia, and a markedly higher prevalence of ischemia [14].

The relationship between CAD and dialysis duration demonstrates the impact of dialysis-related cardiovascular complications, including hemodynamic instability, myocardial stunning, intradialytic hypotension, and chronic volume overload. Researchers have established the link between longer time on dialysis and poorer cardiovascular outcomes. The present research extends this relationship and highlights the need for careful ultrafiltration, proper control of fluid removal, and dry-weight adjustments for patients on long-term dialysis.

Hepatic dysfunction, especially of moderate and severe types, heightened the risk of CAD [15]. In chronic liver disease, systemic inflammation is deregulated, and its effects on the endothelium are further exacerbated by atherosclerosis. Hypoalbuminemia was more frequently present among CAD-positive patients and reflects both liver disease and systemic inflammation. Albumin also indicates nutritional status and is a negative acute-phase reactant. Together, these findings suggest inflammation-driven atherosclerosis. The strong association between elevated CRP and CAD in the current study supports the role of inflammation as a unifying mechanism linking renal dysfunction, hepatic impairment, and coronary disease [16].

In this cohort, higher phosphate levels, elevated calcium-phosphate product, reduced hemoglobin, and lower dialysis adequacy (Kt/V) were observed biochemical differences that further underscore the multifactorial nature of CAD. It is well recognized that ESRD is associated with mineral bone disorder and vascular calcification [17]. In patients with lower Kt/V, inadequate dialysis and therefore poorer clearance of uremic toxins may directly worsen cardiovascular risk.

This study has several limitations. First, its retrospective nature limits the ability to establish causal relationships and may introduce information bias because of reliance on previously recorded medical records. Second, several potentially significant cardiovascular confounders, including smoking status, body mass index, previous cardiovascular events, antiplatelet or statin use, hepatitis B/C infection status, etiology of liver disease, and alcohol consumption, were not uniformly recorded and therefore could not be included in the analysis, which might have resulted in residual confounding. Third, diagnostic heterogeneity may have introduced verification bias because not every patient underwent coronary angiography, which is the gold standard diagnostic method for CAD. In some cases, CAD identification was based on echocardiographic wall motion abnormalities or noninvasive ischemia testing. However, wall motion abnormalities may indicate previous myocardial infarction, cardiomyopathy, or other myocardial disease, and therefore CAD prevalence may be overstated. Lastly, the study was conducted at a single center, which may limit generalizability to broader populations of dialysis patients with different demographic or healthcare profiles.

## Conclusions

It is concluded that coronary artery disease is highly prevalent among patients with end-stage renal disease undergoing maintenance dialysis who also have hepatic dysfunction. This combined disease burden significantly amplifies cardiovascular vulnerability. Older age, diabetes mellitus, longer duration of dialysis, elevated inflammatory markers, and moderate-to-severe hepatic dysfunction were identified as independent predictors of CAD, reflecting the complex interplay between metabolic, inflammatory, and hemodynamic stressors in this population. Future prospective, multicenter studies with standardized angiographic evaluation and longitudinal monitoring of hepatic and inflammatory markers are recommended to better clarify causal pathways. Incorporating advanced imaging and biomarker profiling may further refine cardiovascular risk stratification in this high-risk population.

## References

[1] Barbuto S, Hu L, Abenavoli C (2024). Coronary artery disease in patients undergoing hemodialysis: a problem that sounds the alarm. Rev Cardiovasc Med.

[2] Poli FE, Gulsin GS, McCann GP, Burton JO, Graham-Brown MP (2019). The assessment of coronary artery disease in patients with end-stage renal disease. Clin Kidney J.

[3] Khalil MA, Sadagah NM, Mahmood HH (2025). Prevalence, predictors, and outcomes of coronary artery disease diagnosed by angiography in live-related kidney transplant candidates. BMC Res Notes.

[4] Kumar Karmani J, Ali M (2024). Cardiovascular events and related factors in routine hemodialysis patients with chronic kidney disease (CKD) at a tertiary care hospital in Pakistan. Pak J Health Sci.

[5] Nakić D, Grbić Pavlović P, Vojković M (2024). Coronary artery disease in patients on dialysis: impact of traditional risk factors. Medicina (Kaunas).

[6] Mahmood SN, Hameed N, Naveed HK, Qureshi MF, Imtiaz SH, Rajput AS (2025). Risk factors affecting mortality in Asian hemodialysis patients: a 15-year study from Pakistan. Cureus.

[7] Chandrashekar A, Ramakrishnan S, Rangarajan D (2014). Survival analysis of patients on maintenance hemodialysis. Indian J Nephrol.

[8] Sun Y, Wang Y, Yu W, Zhuo Y, Yuan Q, Wu X (2018). Association of dose and frequency on the survival of patients on maintenance of hemodialysis in China: a Kaplan-Meier and Cox-proportional Hazard Model Analysis. Med Sci Monit.

[9] Kim DH, Park JI, Lee JP (2020). The effects of vascular access types on the survival and quality of life and depression in the incident hemodialysis patients. Ren Fail.

[10] Naylor KL, Kim SJ, McArthur E, Garg AX, McCallum MK, Knoll GA (2019). Mortality in incident maintenance dialysis patients versus incident solid organ cancer patients: a population-based cohort. Am J Kidney Dis.

[11] Cheng XS, VanWagner LB, Costa SP (2022). Emerging evidence on coronary heart disease screening in kidney and liver transplantation candidates: a scientific statement from the American Heart Association: endorsed by the American Society of Transplantation. Circulation.

[12] Mohamed TI, Baqal OJ, Binzaid AA (2020). Outcomes of routine coronary angiography in asymptomatic patients with end-stage renal disease prior to kidney transplantation. Angiology.

[13] Arabi Z, Tawhari MH, Al Rajih HS, Youssouf TM, Abdulgadir MY (2023). Findings of cardiovascular workup of kidney transplant candidates: a retrospective study of a single-center in Saudi Arabia. Int J Nephrol.

[14] Zou Y, Yang M, Wang J (2020). Association of sclerostin with cardiovascular events and mortality in dialysis patients. Ren Fail.

[15] Pokhrel A, Pokhrel BR, Gyawali P (2021). Cardiovascular risk assessment in hemodialysis patients. Nepal Med Coll J.

[16] Saritas T, Floege J (2020). Cardiovascular disease in patients with chronic kidney disease. Herz.

[17] Zoccali C, Mallamaci F, Adamczak M (2023). Cardiovascular complications in chronic kidney disease: a review from the European Renal and Cardiovascular Medicine Working Group of the European Renal Association. Cardiovasc Res.

